# Gene Variants Related to Primary Familial Brain Calcification: Perspectives from Bibliometrics and Meta-Analysis

**DOI:** 10.1523/ENEURO.0058-25.2025

**Published:** 2025-06-18

**Authors:** Dehao Yang, Yangguang Lu, Honghao Huang, Yiqun Chen, Zihan Jiang, Ruotong Yao, Yiran Bu, Yu Li, Zhidong Cen, Wei Luo

**Affiliations:** ^1^Department of Neurology, The Second Affiliated Hospital, Zhejiang University School of Medicine, Hangzhou 310009, China; ^2^The First School of Medicine, School of Information and Engineering, Wenzhou Medical University, Wenzhou 325035, China; ^3^Department of Cardiology, Renji Hospital, Shanghai Jiao Tong University School of Medicine, Shanghai 200127, China; ^4^The Second School of Medicine, Wenzhou Medical University, Wenzhou 325035, China

**Keywords:** bibliometrics, Fahr’s disease, gene variants, meta-analysis, primary familial brain calcification

## Abstract

The genetic role and specific effects of primary familial cerebral calcification (PFBC) are still unclear. We aim to analyze bibliometric features in studies related to PFBC, investigate variant detection rates in patients with brain calcifications, and examine the phenotypic characteristics of PFBC patients. A comprehensive search of studies on the genetic effects of PFBC up until December 31, 2024, was conducted across Web of Science, PubMed, Embase, and Scopus. A random-effects meta-analysis combined variant detection rates for genes *SLC20A2*, *PDGFRB*, *PDGFB*, *XPR1*, *MYORG*, *JAM2*, *CMPK2*, and *NAA60*. Data on total calcification scores (TCS), age of onset, and the prevalence of various phenotypes in PFBC patients were also aggregated. Publication bias was assessed using Egger's linear regression, and a leave-one-out sensitivity analysis was performed. Of 1,267 records, 224 were included in the bibliometric analysis. Keywords “primary familial brain calcification” and “*SLC20A2*” were most prominent. Eighteen articles were included in the meta-analysis, revealing higher variant rates for *SLC20A2* (16.7%, 95% CI: 10.0–24.6) and *MYORG* (16.8%, 95% CI: 0.0–54.0), which were associated with higher TCS. The average age of onset was 43.69 years (95% CI: 36.17–51.21). Cognitive impairment (45.3%, 95% CI: 35.7–55.1) and psychiatric symptoms (30.8%, 95% CI: 17.2–46.2) had relatively higher prevalence rates. No significant publication bias was found (*p* > 0.05), and the sensitivity analysis confirmed the results’ robustness. *SLC20A2* and *MYORG* variants had higher detection rates, with cognitive impairment and psychiatric symptoms being common in PFBC patients. Continued research is essential to further explore these genetic variants.

## Significance Statement

This study reveals that *SLC20A2* and *MYORG* gene variants are key drivers of primary familial brain calcification (PFBC), a neurodegenerative disorder marked by brain calcium deposits. Using global data, we show these variants correlate with severe calcification and frequently manifest as cognitive decline or psychiatric symptoms, while nearly a quarter of patients remain asymptomatic. By integrating genetic and clinical analyses, we provide the first systematic comparison of PFBC-associated genes and phenotypes, offering critical insights for diagnosis, genetic counseling, and mechanistic research. These findings highlight the need for expanded screening of understudied genes and global collaborations to address gaps in understanding this underdiagnosed condition, ultimately guiding therapeutic strategies for affected individuals.

## Introduction

Primary familial brain calcification (PFBC) is a rare neurodegenerative disorder characterized by bilateral brain calcifications in the basal ganglia. In 1974, the term “idiopathic basal ganglia calcification” was first introduced to describe two cases of familial basal ganglia calcification, which presented with characteristics of “dystonic deformities” and unexplained brain calcification (Caraceni et al., 1974). Over the following decades, studies on familial clusters suggested a potential genetic etiology for this idiopathic basal ganglia calcification (Foley, 1951; Boller et al., 1977; Ellie et al., 1989). It was not until 2012, when the first dominant genetic factor, *SLC20A2*, was identified (Wang et al., 2012), that the genetic basis of PFBC was firmly established. PFBC is typically inherited in an autosomal dominant pattern (Guo et al., 2019), and to date, four dominant pathogenic genes have been identified: *SLC20A2* (Wang et al., 2012), *PDGFRB* (Nicolas et al., 2013b), *PDGFB* (Keller et al., 2013), and *XPR1* (Legati et al., 2015). In recent years, four recessive pathogenic genes, *MYORG* (Yao et al., 2018), *JAM2* (Cen et al., 2020), *CMPK2* (Zhao et al., 2022), and *NAA60* (Chelban et al., 2024), have also been discovered. Among these, the role of *CMPK2* in PFBC pathogenesis has not been independently replicated, and its potential as a causative gene remains under debate.

The clinical phenotype of PFBC is heterogeneous, encompassing motor disturbances, neuropsychiatric symptoms, and, in a significant proportion of cases, the absence of obvious symptoms (Manyam et al., 2001; Nicolas et al., 2015; Tadic et al., 2015). Disease onset can occur at any age, although more than half of patients typically develop symptoms between the ages of 30 and 50 years (Nicolas et al., 2015). Currently, PFBC diagnosis is supported by the detection of brain calcifications through computed tomography (CT) scans, with confirmation through the identification of pathogenic variants in one of these eight PFBC-associated genes (Legati et al., 2015; Westenberger et al., 2019). While traditionally considered rare, recent population-based genomic analyses suggest PFBC is more common than previously thought, though frequently underdiagnosed (Nicolas et al., 2018). Epidemiological inference based on genetic screening suggests an overall prevalence of PFBC of up to 0.66% (Chen et al., 2019). Therefore, the impact of PFBC on global health needs to be further emphasized.

Previous studies have indicated that among the known PFBC-associated genes, variants in *SLC20A2* are the most common, accounting for ∼40% of familial cases (David et al., 2016). *SLC20A2* encodes the transmembrane sodium-inorganic phosphate cotransporter PiT-2, which plays a crucial role in phosphate clearance from cerebrospinal fluid (Jensen et al., 2016). Additionally, *XPR1* is also implicated in phosphate transport, with functional studies showing that variants in *XPR1* severely impair its membrane localization and phosphate efflux activity (Legati et al., 2015; Anheim et al., 2016). The loss of *NAA60* function may also reduce extracellular phosphate uptake by impairing the cell membrane expression of the *SLC20A2* gene (Chelban et al., 2024). In addition to pathophysiological factors related to phosphate metabolism disorders, genes encoding platelet-derived growth factor receptor β (PDGF-Rβ) and its ligand (*PDGFB* and *PDGFRB*), as well as recessive pathogenic genes *MYORG* and *JAM2*, may compromise blood–brain barrier (BBB) integrity by damaging the neurovascular unit (NVU). Increased BBB permeability could allow high concentrations of phosphate in the blood to infiltrate the brain, ultimately contributing to calcification (Xu et al., 2023). Given the rapid progress in PFBC research and its associated pathogenic genes, understanding trends and advancements in this field is crucial.

However, there remains a lack of systematic assessments and comparisons regarding the variant detection rates, severity, and age of onset of different PFBC-related pathogenic genes in patients with brain calcifications. Furthermore, there is insufficient evaluation of the prevalence of clinical phenotypes in PFBC patients, as well as a comprehensive review of past research on PFBC and its associated pathogenic genes. Therefore, this study aims to address these gaps in the literature by conducting a meta-analysis, incorporating bibliometric perspectives, to identify key research trends and hotspots in PFBC, as well as to determine the variant detection rates of PFBC-related genes in patients with brain calcifications and the phenotypic characteristics of PFBC patients.

## Materials and Methods

### Search strategy

Two researchers systematically searched studies published up until December 31, 2024, in PubMed, Embase, Scopus, and Web of Science databases, with no language restrictions. The search was performed using the following keyword combination: (“calcification” OR PFBC OR IBGC OR “Fahr disease”) AND (*SLC20A2* OR *PDGFRB* OR *PDGFB* OR *XPR1* OR *MYORG* OR *JAM2* OR *CMPK2* OR *NAA60*). References from relevant systematic or narrative reviews identified in the search were also screened for additional studies. Given the need for citation databases in bibliometric analysis, and the inability to integrate other databases (Archambault et al., 2009; Aria and Cuccurullo, 2017), the bibliometric analysis was limited to studies indexed in the Web of Science Core Collection (WOSCC), specifically the Science Citation Index Expanded (SCIE) and the Social Sciences Citation Index (SSCI). Bibliometric data were exported as “plain text” files containing full records and references.

### Selection criteria

For bibliometric analysis, we included all studies from the WOSCC database related to the pathogenic genes of PFBC with available bibliometric data. Studies such as letters, conference abstracts, corrigenda, and editorials were excluded.

For the meta-analysis, we included studies that performed gene sequencing and screening in patients with brain calcifications identified by imaging features, reporting variants in at least one of the following genes: *SLC20A2*, *PDGFRB*, *PDGFB*, *XPR1*, *MYORG*, *JAM2*, *CMPK2*, and *NAA60*. Studies were excluded if they met any of the following criteria: (1) animal studies; (2) case reports, series with fewer than 15 cases, or reviews; (3) duplicate studies from the same population; and (4) missing or uncalculable key outcome data. When multiple publications reported on the same population, we included the publication with the largest sample size, longest follow-up period, or most comprehensive study outcomes. The studies were selected independently by two researchers, with disagreements resolved by consulting a third author.

### Bibliometric analysis

We recorded information including the title, institution, source (journal or book), authors, references, subject area, and keywords using Microsoft Excel. Analysis was performed using the “bibliometrix” package in R version 4.3.1 (Aria and Cuccurullo, 2017) and CiteSpace software (version 6.2.R2 Basic). Indicators such as Total Citation (TC), Local Citation (LC), Normalized Total Citation (NTC), Normalized Local Citation (NLC), Average Article Citation (AAC), Articles Fractionalized (AF), and the H-index were applied to evaluate the impact and research output of countries, journals, authors, or studies.

### Meta-analysis

The meta-analysis was conducted following the Meta-analysis of Observational Studies in Epidemiology (MOOSE) guidelines (Stroup et al., 2000). Two researchers independently assessed study eligibility and extracted relevant data from all included studies, including the title, first author's name, publication year, country or region of the study population, sample size, study type, gene sequencing method, number of screened patients, genes screened, number of patients with identified gene variants, total calcification score (TCS), age at disease onset, disease phenotypes, and the number of occurrences for each phenotype. Any discrepancies in data extraction were resolved through consensus.

The methodological quality of cross-sectional studies was assessed using the Joanna Briggs Institute (JBI) Checklist (Zeng et al., 2015), which evaluates eight dimensions, each rated as Yes, Unclear, No, or Not Applicable. A study was considered high quality if at least six “Yes” responses were obtained with no “No” responses. Additionally, the Newcastle-Ottawa Scale (NOS; Stang, 2010) was used to assess the methodological quality of cohort and case–control studies, with a score of ≥7 considered high quality. A follow-up period longer than 2 years was considered sufficiently long. All quality assessments were performed independently by two researchers, with discrepancies resolved through consensus and the involvement of a third researcher.

The primary outcome of this study was the variant detection rate of variants in *SLC20A2*, *PDGFRB*, *PDGFB*, *XPR1*, *MYORG*, *JAM2*, *CMPK2*, and *NAA60* in patients with brain calcifications. The variant rate was calculated based on unrelated families and probands, with no repeated participants. Secondary outcomes included the age of onset, severity of brain calcification, and the prevalence of phenotypes in PFBC patients. The severity of brain calcification was assessed using the TCS score. Our classification of disease phenotypes was based on the descriptions reported in the included studies and was divided into Headache, Cognitive Disorder, Movement Disorder, Mental Symptom, Dysarthria, and Asymptomatic. These phenotypes were accurately defined in the original studies, from which detailed information can be obtained. We used a random-effects model to pool outcome data and calculate 95% confidence intervals (CIs). Heterogeneity between studies was assessed using Cochrane's Q test and the I^2^ statistic, with I^2^ values exceeding 50 and 75% considered moderate and high heterogeneity, respectively (Higgins et al., 2003). For outcomes related to the severity of brain calcification, we performed subgroup analyses based on the mutated gene and used the *Z*-test to assess intergroup differences. Egger's linear regression test was used to evaluate publication bias (Egger et al., 1997). A Baujat plot was employed to explore sources of heterogeneity further (Baujat et al., 2002). Sensitivity analysis was conducted using the “leave-one-out” method to assess the robustness of the pooled results (Deeks et al., 2019). In all statistical analyses, a *p* value of <0.05 was considered statistically significant. Statistical analyses for the meta-analysis were performed using the meta package in R version 4.3.1.

## Results

### Bibliometric characteristics

**Figure 1.**
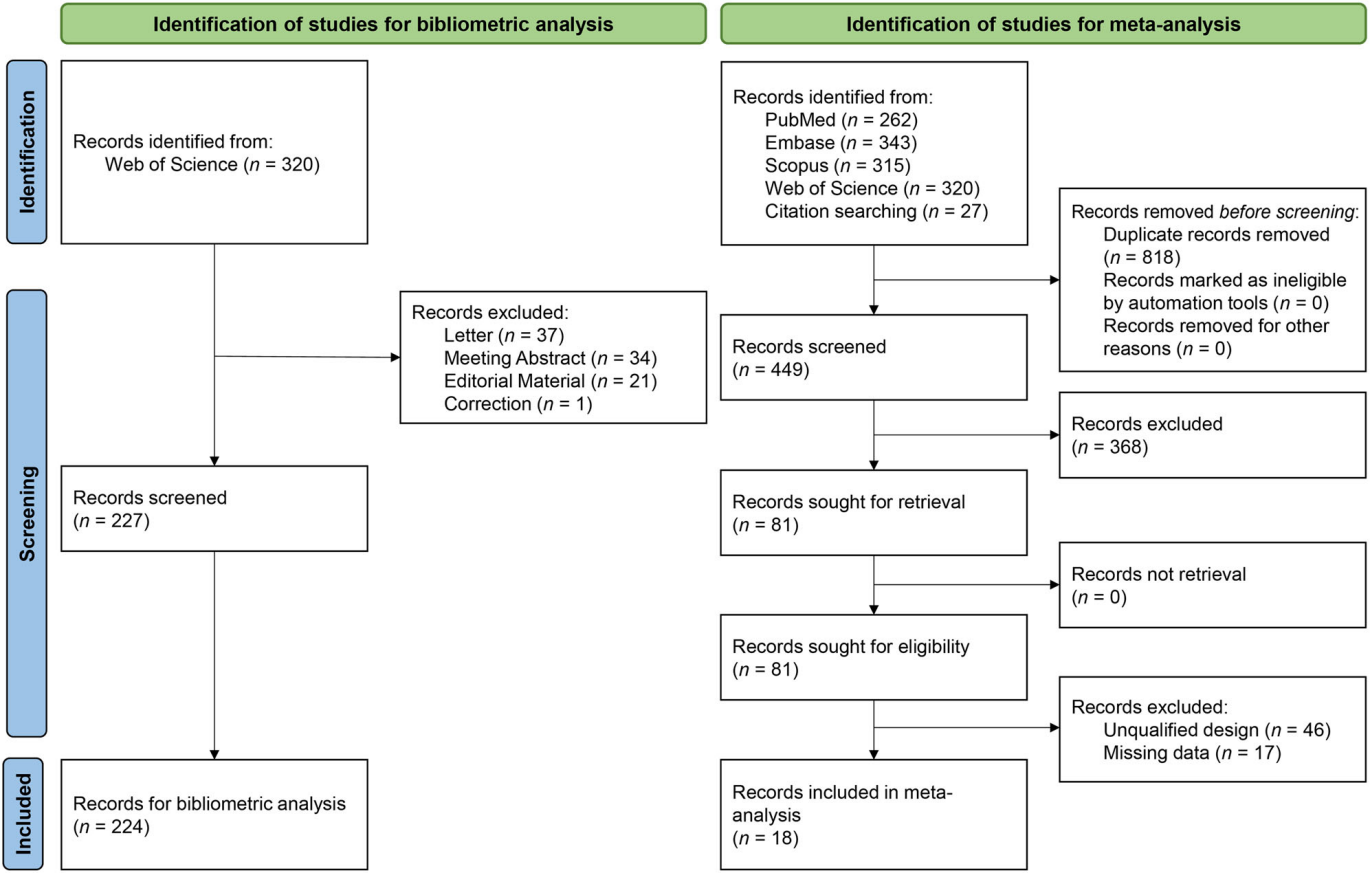
Flow diagram including reasons for exclusion of full-text articles.

A total of 320 publications were identified through the WOSCC database. After excluding 96 studies that did not meet the inclusion criteria, 224 studies were included in the bibliometric analysis (Fig. 1). The publications spanned the years 2012–2024. Among these studies, 193 were original articles (86.16%) and 31 were reviews (13.84%), involving 1,551 authors, 400 keywords, 4,168 references, and 113 sources (journals or books). On average, each article was cited 24.82 times, and each article was written by 10.5 authors. International collaborative research accounted for 28.12% of the total publications. The average annual growth rate of publications in this field was 18.06%.

**Figure 2.**
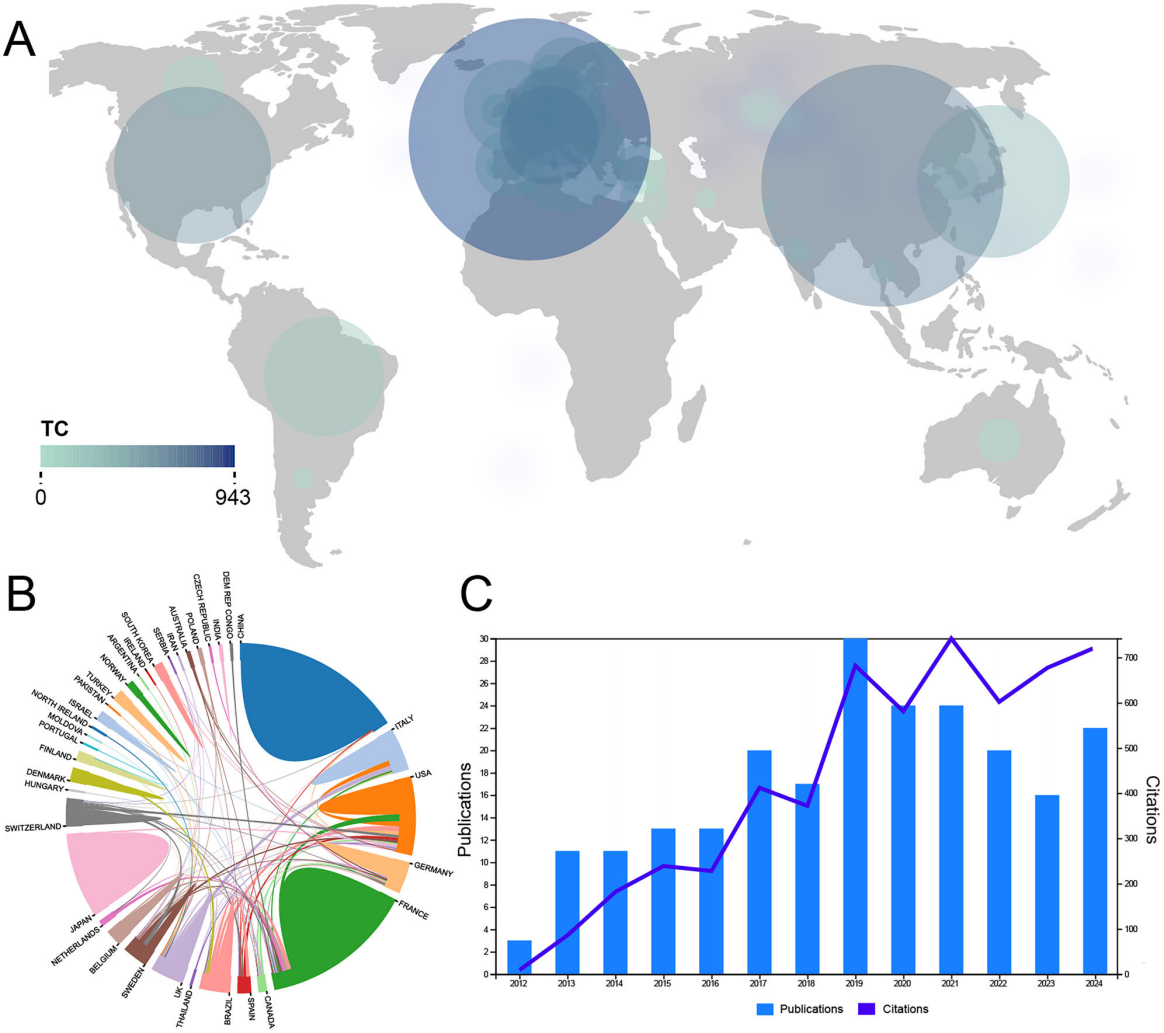
Information of regions on relevant research. ***A***, Distribution of regions contributing to the field. ***B***, Regions’ collaboration world map. ***C***, Trends in the number of publications and citations.

In the research area of PFBC-related gene variants, publication frequency analysis, including co-authors, revealed that China and France had the highest publication rates, while there was a notable lack of research from the Arabian Peninsula and Africa. Additionally, studies from France had the highest citation rates (Fig. 2*A*). The analysis of interregional collaborations showed that the highest collaboration frequencies occurred between the United States, Brazil, and France, highlighting the dominant role of the United States in international cooperation (Fig. 2*B*). Driven by global research efforts, the number of publications and citations in this field has shown a steady increase year by year (Fig. 2*C*).

**Figure 3.**
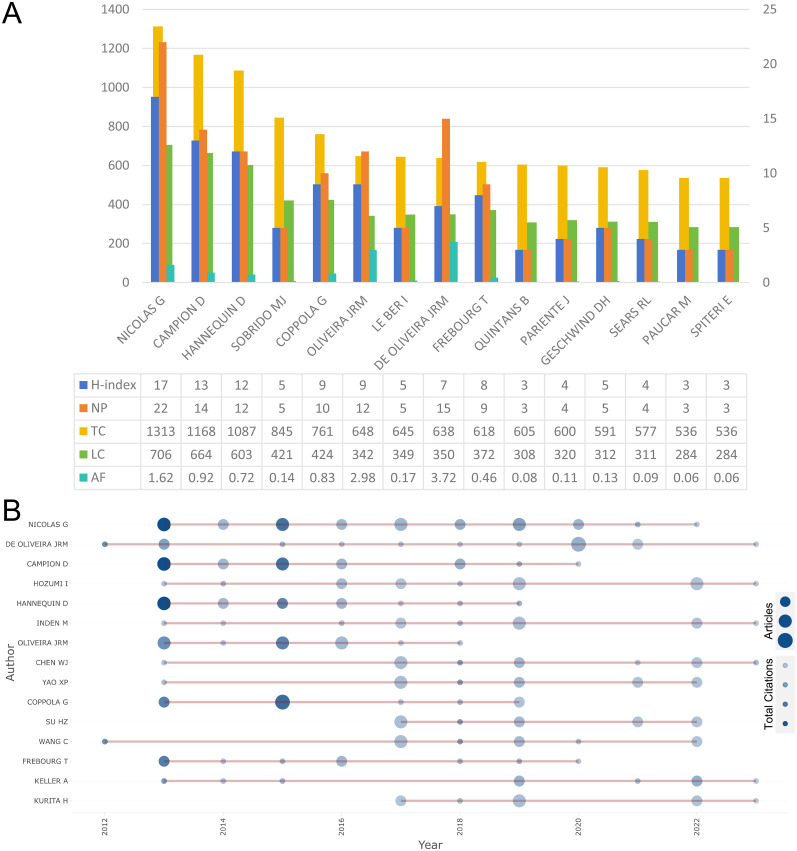
Bibliometric analysis of authors: (***A***) plots of the top 20 most produced authors’ H-index and citation data; (***B***) authors’ production over time of the top 10 most productive authors. NP, number of publications; AF, articles fractionalized; TC, total citation; LC, local citation.

The study by Wang et al. (2012) in 2012, titled “Variants in *SLC20A2* link familial idiopathic basal ganglia calcification with phosphate homeostasis,” had the highest TC and LC. In contrast, the 2018 study by Yao et al. (2018) in *Neuron*, titled “Biallelic variants in *MYORG* cause autosomal recessive primary familial brain calcification”, had the highest NTC and NLC. These two studies are the most influential in the field, representing the discovery of the first dominant and recessive genetic variants associated with PFBC, respectively, and have made significant contributions to research on PFBC-related genetic variants (Table 1). Dr. Nicolas has the highest h-index, publication volume, TC, and LC, indicating the greatest influence in this field. The top 20 most influential authors maintain a relatively stable annual publication rate, with articles published in 2013 and 2015 receiving the most citations (Fig. 3). Regarding the sources of publications, the *Journal of Molecular Neuroscience* published the largest number of articles on PFBC, while *Nature Genetics* had the highest TC and *Scientific Reports* had the highest h-index. Among all source journals, the 11 journals with the highest publication volumes, including the *Journal of Molecular Neuroscience*, were identified as core journals according to Bradford's Law (Weinstock, 1971).

**Table 1.** Top 10 most cited publications on PFBC-related researches

SCR	Title	FA	Year	Journal	TC	LC	TCY	NTC	NLC
1	Mutations in *SLC20A2* link familial idiopathic basal ganglia calcification with phosphate homeostasis	Wang C.	2012	Nat Genet	323	148	26.92	2.26	2.98
2	Mutations in the gene encoding *PDGF-B* cause brain calcifications in humans and mice	Keller A.	2013	Nat Genet	256	127	23.27	2.64	2.43
3	Mutations in *XPR1* cause primary familial brain calcification associated with altered phosphate export	Legati A.	2015	Nat Genet	231	111	25.67	3.69	3.81
4	Mutation of the *PDGFRB* gene as a cause of idiopathic basal ganglia calcification	Nicolas G.	2013	Neurology	226	124	20.55	2.33	2.37
5	Phenotypic spectrum of probable and genetically-confirmed idiopathic basal ganglia calcification	Nicolas G.	2013	Brain	173	95	15.73	1.79	1.82
6	Biallelic mutations in *MYORG* cause autosomal recessive primary familial brain calcification	Yao X.P.	2018	Neuron	122	75	20.33	3.73	5.93
7	Role of phosphate sensing in bone and mineral metabolism	Chande S.	2018	Nat Rev Endocrinol	122	5	20.33	3.73	0.40
8	Mutations in *SLC20A2* are a major cause of familial idiopathic basal ganglia calcification	Hsu S.C.	2013	Neurogenetics	117	74	10.64	1.21	1.42
9	Novel overgrowth syndrome phenotype due to recurrent de novo *PDGFRB* mutation	Takenouchi T.	2015	J Pediatr	85	12	9.44	1.36	0.41
10	Loss of function of *SLC20A2* associated with familial idiopathic Basal Ganglia calcification in humans causes brain calcifications in mice	Jensen N.	2013	J Mol Neurosci	83	48	7.55	0.86	0.92

SCR, standard competition ranking; FA, first author; LC, local citation; TC, total citation; TCY, total citation per year; NLC, normalized local citations; NTC, normalized total citation.

### Co-occurrence and co-citation statistics

**Figure 4.**
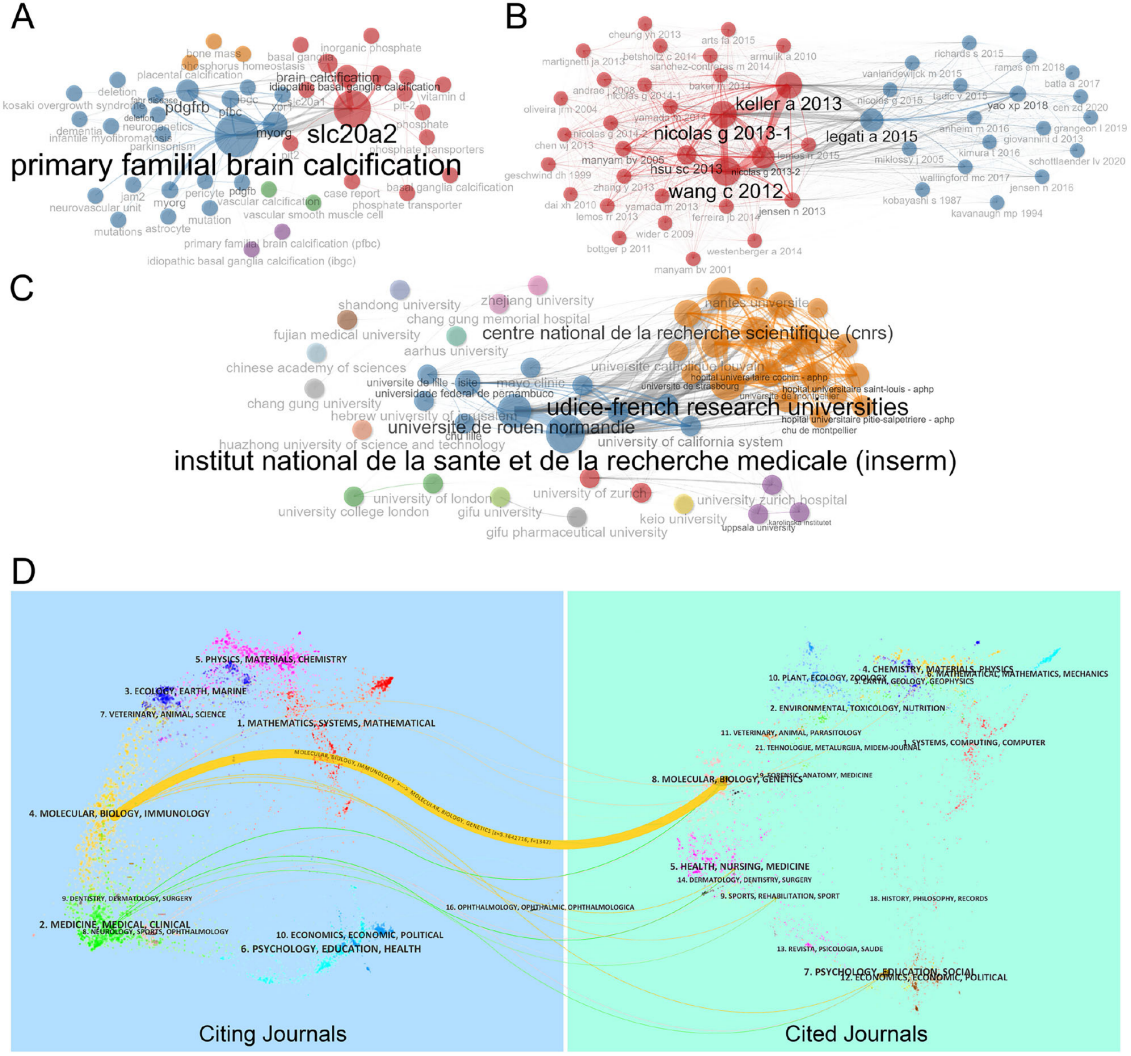
Co-occurrence and network analysis. ***A***, Network analysis of the keywords. ***B***, The co-citation relationship of the articles. ***C***, Network of collaborative relationships between institutions. ***D***, Journal analysis in the research field of genetic effects in PFBC. The dual-map overlay of journals on PFBC generated by CiteSpace software. Specifically, the labels represented different research subjects covered by the journals. Different colored lines correspond to the different paths of references, starting from the citing journals (left half) to the cited journals (right half). The main citing journals were shown in the blue box and the main cited journals were shown in the green box.

In the co-occurrence network analysis of keywords, all keywords were divided into five major clusters. Among these, “primary familial brain calcification” and “*SLC20A2*” had the largest node sizes and betweenness centrality, indicating the strongest co-occurrence and making them the most important keywords. Notably, other related genes, such as “*PDGFRB*,” “*MYORG*,” “*XPR1*,” and “*JAM2*,” as well as phenotypic traits like “dementia” and “parkinsonism,” also exhibited significant node sizes (Fig. 4*A*). In the co-citation network analysis of all studies, articles were divided into two major clusters. The articles by Nicolas, Keller, and Wang had the largest node sizes, indicating strong co-citation relationships between these studies (Fig. 4*B*).

In the network analysis of institutional collaborations, the institution with the highest activity in related research was the French National Institute of Health and Medical Research (INSERM). The highest co-occurrence rate was observed between INSERM and the University of Rouen Normandy, indicating broad collaboration between these two institutions. Additionally, Zhejiang University in China exhibited the highest betweenness centrality, while Gifu Pharmaceutical University and Gifu University in Japan had the highest closeness centrality, making them key institutions in facilitating collaboration within the field (Fig. 4*C*).

The dual-map overlay analysis of citing and cited journals revealed the disciplinary distribution of these journals. The left half of the map represents citing journals, while the right half represents cited journals. The colored lines represent citations and show the relationship between citing and cited journal articles. The results indicated that one of the primary citation pathways in PFBC-related research is from Molecular Biology & Genetics (cited journals) to Molecular Biology & Immunology (citing journals), highlighting the continued focus on basic research. “Genetics,” “Immunology,” and “Molecular Biology” remain core areas of focus, while the clinical translational significance of PFBC-related research requires further exploration (Fig. 4*D*).

### Study characteristics of meta-analysis

Based on the bibliometric analysis, a total of 1,267 records were identified through database and manual searches. After determining the eligibility of the studies for full-text review, 81 studies were assessed, and following a detailed evaluation, 18 studies were included in the meta-analysis (Fig. 1). These studies, published between 2013 and 2021, spanned six regions, five PFBC-related genes, and six types of PFBC-related clinical phenotypes (Table 2). A quality assessment of the methodologies of the 12 cross-sectional studies (Chen et al., 2013; Baker et al., 2014; Sanchez-Contreras et al., 2014; Nicolas et al., 2015; Anheim et al., 2016; David et al., 2016; Gebus et al., 2017; Ramos et al., 2018; Giorgio et al., 2019; Grangeon et al., 2019; Chelban et al., 2020; Kurita et al., 2021), two case–control studies(Nicolas et al., 2013a; Hozumi et al., 2018), and four cohort studies(Hsu et al., 2013; Chen et al., 2019, 2020; Guo et al., 2019) included in the meta-analysis was performed (Table 3). All included studies were of high quality, with two case–control studies receiving a score of 9 on the NOS scale.

**Table 2.** Characteristics of the 18 included in meta-analysis

Author	Year	Country	Study Design	Sample Size	Reported Gene	Reported Phenotype
Chen et al.	2013	China	CS	20 (14 Familial + 6 Sporadic)	*SLC20A2*	Headache, movement disorder, dysarthria, asymptomatic
Nicolas et al.	2013	France	CC	72 (19 Familial + 6 Sporadic + 47 Probable PFBC)	*SLC20A2, PDGFRB*	Cognitive disorder, movement disorder, mental symptom, asymptomatic
Hsu et al.	2013	USA	RC	218 (All Familial)	*SLC20A2*	N.A.
Baker et al.	2014	USA	CS	27 (All Familial)	*SLC20A2*	N.A.
Sanchez et al.	2014	USA	CS	26 (All Familial)	*PDGFRB, PDGFB*	N.A.
Nicolas et al.	2015	France	CS	57 (46 Familial + 11 Sporadic)	*SLC20A2, PDGFRB, PDGFB*	Cognitive disorder, movement disorder, mental symptom, asymptomatic
Anheim et al.	2016	France	CS	18 (All Sporadic)	*XPR1*	Movement disorder
David et al.	2016	France	CS	24 (14 Familial + 10 Sporadic)	*SLC20A2*	headache, cognitive disorder, mental symptom
Gebus et al.	2017	France	CS	80 (All Sporadic)	*SLC20A2, PDGFRB, PDGFB, XPR1*	Cognitive disorder, movement disorder
Ramos et al.	2018	Italy	CS	177 (All Familial)	*SLC20A2, PDGFRB, PDGFB, XPR1*	Cognitive disorder, movement disorder, mental symptom, asymptomatic
Hozumi et al.	2018	Japan	CC	42 (29 Familial + 13 HC)	*SLC20A2, PDGFB*	N.A.
Chen et al.	2019	China	RC	273 (All Familial)	*SLC20A2, PDGFRB, PDGFB, XPR1*	Headache, movement disorder
Chen et al.	2020	China	RC	435 (All Familial)	*MYORG*	Movement disorder, dysarthria, mental symptom
Giorgio et al.	2019	Italy	CS	50 (All Sporadic)	*SLC20A2*	Headache, movement disorder, mental symptom, asymptomatic
Grangeon et al.	2019	France	CS	29 (All Familial)	*MYORG*	Cognitive disorder, asymptomatic
Guo et al.	2019	China	RC	226 (35 Familial + 191 Sporadic)	*SLC20A2, PDGFRB, PDGFB, XPR1*	headache, movement disorder, asymptomatic
Chelban et al.	2020	UK	CS	86 (All Familial)	*MYORG*	Headache, cognitive disorder, movement disorder, dysarthria, asymptomatic
Kurita et al.	2021	Japan	CS	173 (All Sporadic)	*SLC20A2, PDGFRB*	Headache

CS, cross-sectional study; CC, case–control study; RC, retrospective cohort study; HC, healthy control; Ref, reference.

**Table 3.** Methodological quality evaluation of the 18 studies included in the meta-analysis

Study	Major components of JBI checklist for cross-sectional studies								
	Were the criteria for inclusion in the sample clearly defined?	Were the study subjects and the setting described in detail?	Was the exposure measured in a valid and reliable way?	Were objective criteria used for measurement of the condition?		Were confounding factors identified?	Were strategies to deal with confounding factors stated?	Were the outcomes measured in a valid and reliable way?	Was appropriate statistical analysis used?
Chen et al. (2013)	Yes	Yes	Yes	Unclear		Yes	Unclear	Yes	Yes
Baker et al. (2014)	Yes	Yes	Yes	Yes		Unclear	Unclear	Yes	Yes
Sanchez-Contreras et al. (2014)	Yes	Yes	Yes	Yes		Unclear	Unclear	Yes	Yes
Nicolas et al. (2015)	Yes	Yes	Yes	Yes		Yes	Yes	Yes	Yes
Anheim et al. (2016)	Yes	Yes	Yes	Yes		Unclear	Unclear	Yes	Yes
David et al. (2016)	Yes	Yes	Yes	Yes		Yes	Yes	Yes	Yes
Gebus et al. (2017)	Yes	Yes	Yes	Yes		Yes	Yes	Yes	Yes
Ramos et al. (2018)	Yes	Yes	Yes	Yes		Yes	Unclear	Yes	Yes
Giorgio et al. (2019)	Yes	Yes	Yes	Yes		Unclear	Unclear	Yes	Yes
Grangeon et al. (2019)	Yes	Yes	Yes	Yes		Yes	Yes	Yes	Yes
Chelban et al. (2020)	Yes	Yes	Yes	Yes		Yes	Yes	Yes	Yes
Kurita et al. (2021)	Yes	Yes	Yes	Yes		Yes	Yes	Yes	Yes
Study	Items of Newcastle-Ottawa Scale for case–control studies								
	Selection				Comparability	Exposure			Total
	(1)	(2)	(3)	(4)		(1)	(2)	(3)	
Nicolas et al. (2013a)	★	★	★	★	★★	★	★	★	9
Hozumi et al. (2018)	★	★	★	★	★★	★	★	★	9
Study	Items of Newcastle-Ottawa Scale for cohort studies								
	Selection				Comparability	Outcome			Total
	(1)	(2)	(3)	(4)		(1)	(2)	(3)	
Hsu et al. (2013)	★	★	★	★	★★	★	✩	✩	7
Chen et al. (2020)	★	★	★	★	★★	★	★	✩	8
Chen et al. (2019)	★	★	★	★	★★	★	★	✩	8
Guo et al. (2019)	★	★	★	★	★✩	★	★	✩	7

NOS scare for case–control studies: selection, (1) is case definition adequate, (2) representativeness of the cases, (3) selection of controls, (4) definition of controls; comparability, comparability on basis of design or analysis; exposure, (1) ascertainment of exposure, (2) same method of ascertainment for cases and controls, (3) nonresponse rate. NOS scale for cohort studies: Selection, (1) representativeness of the exposed cohort, (2) selection of the nonexposed cohort, (3) ascertainment of exposure to implants, (4) demonstration that outcome of interest was not present at start of study; comparability: comparability of cohorts on the basis of the design or analysis; outcome, (1) assessment of outcome, (2) was follow-up long enough for outcomes to occur, (3) adequacy of follow-up cohorts.

### Detection rate of PFBC-related gene variants

**Figure 5.**
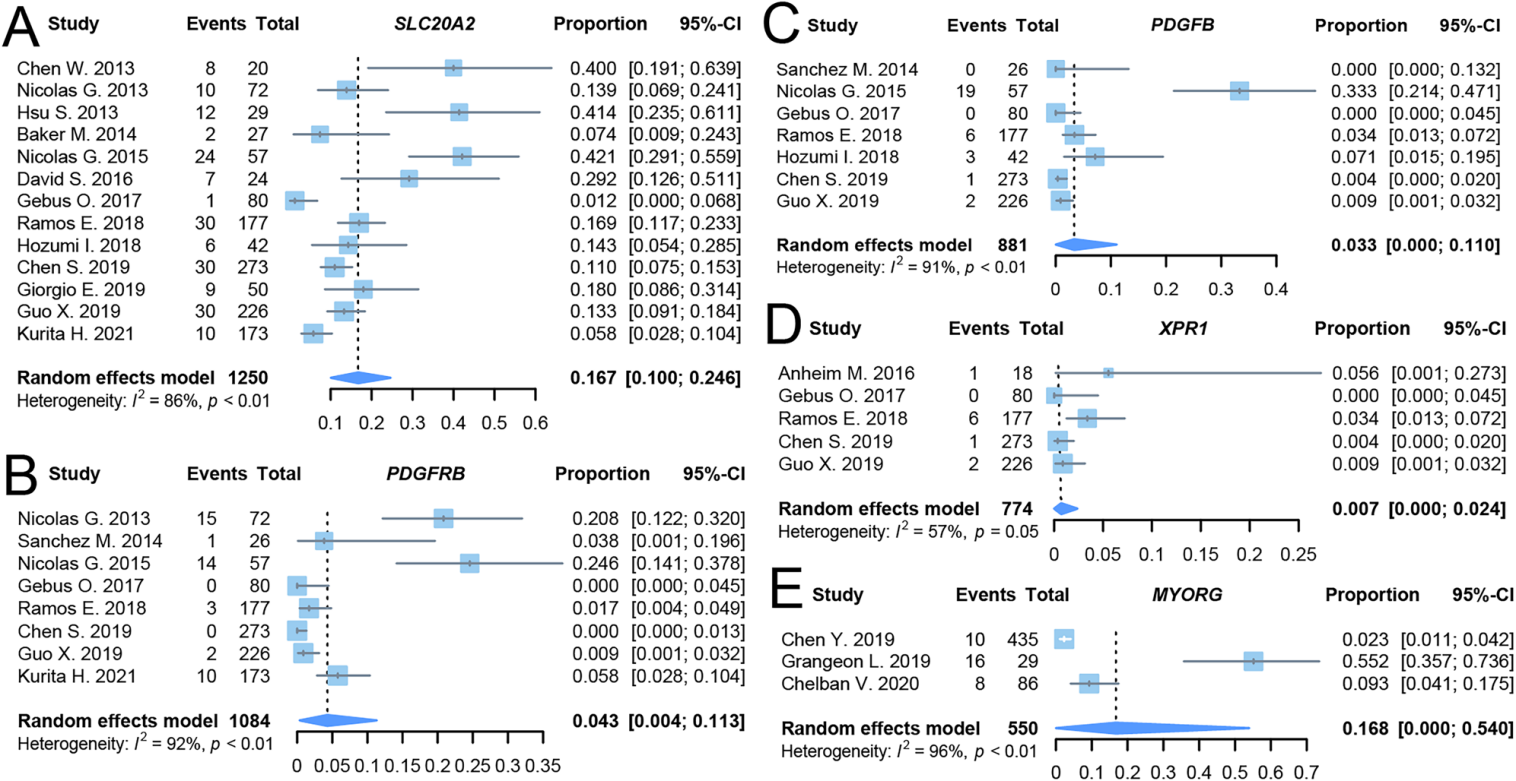
Forest plots of primary outcome in meta-analysis. ***A***, *SLC20A2* variant detection rate combined result. ***B***, *PDGFRB* variant detection rate combined result. ***C***, *PDGFB* variant detection rate combined result. ***D***, *XPR1* variant detection rate combined result. ***E***, *MYORG* variant detection rate combined result.

Thirteen studies screened for variants in the *SLC20A2* gene region. The random-effects model indicated that 16.7% (95% CI: 10.0–24.6, *I*^2^ = 86%) of patients exhibited genetic variants in the *SLC20A2* gene region. Eight studies screened the *PDGFRB* gene region, finding 4.3% (95% CI: 0.4–11.3, *I*^2^ = 92%) of patients with variants in this region. Seven studies focused on the *PDGFB* gene region, with 3.3% (95% CI: 0.0–11.0, *I*^2^ = 91%) of patients exhibiting variants. Five studies analyzed the *XPR1* gene region, reporting a variant rate of 0.7% (95% CI: 0.0–2.4, *I*^2^ = 57%). Three studies assessed the *MYORG* gene region, revealing a variant rate of 16.8% (95% CI: 0.0–54.0, *I*^2^ = 96%; Fig. 5). Egger's linear regression analysis suggested no significant publication bias (*p* > 0.05).

Except for the five studies reporting variants in the *XPR1* gene, which exhibited moderate heterogeneity, the other four outcomes displayed high heterogeneity. The Baujat plot indicated that the studies by Gebus et al. (2017), Chen et al. (2019), Nicolas et al. (2015), and four studies by Chen et al. (2019) were the primary sources of heterogeneity for the studies on *SLC20A2*, *PDGFRB*, *PDGFB*, and *MYORG*, respectively. However, sensitivity analysis using the “leave-one-out” method showed that the combined results were robust, as excluding any single study did not significantly alter the outcomes.

### Phenotypic characteristics of PFBC patients

**Figure 6.**
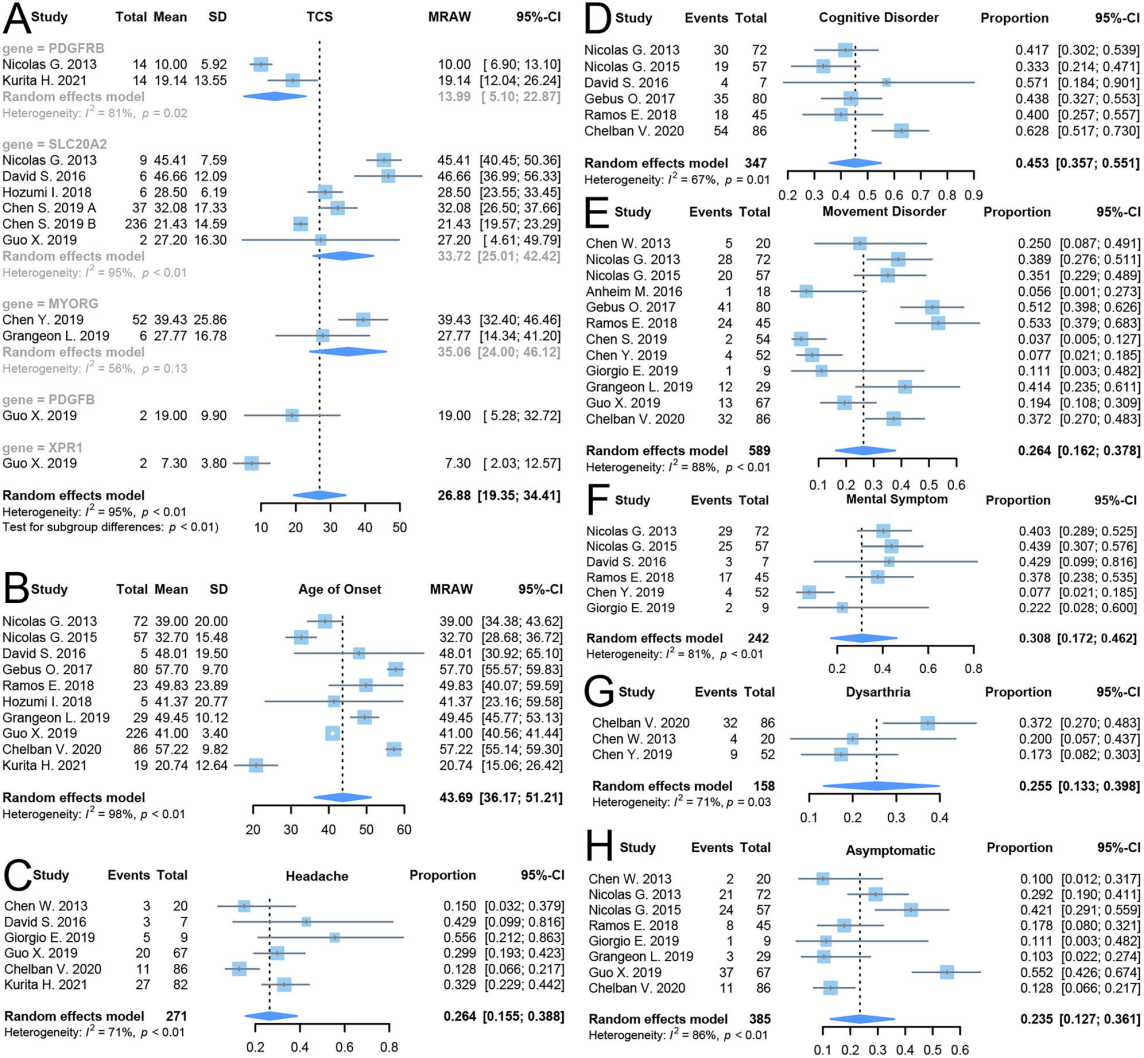
Forest plot of secondary outcome in meta-analysis. ***A***, Differences among TCS scores of variants’ location; ***B***, combined result of age of onset. ***C***, Combined results of headache phenotype rate. ***D***, Combined results of cognitive disorder phenotype rate. ***E***, Combined results of movement disorder phenotype rate. ***F***, Combined results of mental symptom phenotype rate. ***G***, Combined results of language disorder phenotype rate. ***H***, Combined results of asymptomatic phenotype rate.

Eight studies reported TCS scores for PFBC patients. The random-effects model showed that patients with variants in the *PDGFB* gene region had an average TCS score of 13.99 (95% CI: 5.10–22.87, *I*^2^ = 81%), while patients with variants in the *SLC20A2* gene region had an average TCS score of 33.72 (95% CI: 25.01–42.42, *I*^2^ = 95%). Patients with variants in the *MYORG* gene region had an average TCS score of 35.06 (95% CI: 24.00–46.12, *I*^2^ = 56%). *Z*-test results showed significant differences in TCS scores between different genes (*p* < 0.01), with patients harboring variants in the *XPR1* (7.30, 95%CI: 2.03–12.57) and *PDGFRB* (13.99, 95%CI: 5.10–22.87) region exhibiting lower TCS scores compared with those with variants in other genes, although only one study reported TCS score data for the *XPR1* gene and thus the random-effects model could not be used for pooling. Except for the *MYORG* gene region, which showed moderate heterogeneity, the other studies demonstrated considerable heterogeneity (Fig. 6*A*). Additionally, 10 studies reported the onset age of PFBC symptoms. The random-effects model indicated that the average age of onset for PFBC patients was 43.69 years (95% CI: 36.17–51.21, *I*^2^ = 98%; Fig. 6*B*).

In the meta-analysis of various clinical phenotypes in PFBC patients, the random-effects model indicated the following prevalence rates: headache occurred in 26.4% (95% CI: 15.5–38.8, *I*^2^ = 71%) of patients (Fig. 6*C*); cognitive impairment was present in 45.3% (95% CI: 35.7–55.1, *I*^2^ = 67%; Fig. 6*D*); movement disorders were observed in 26.4% (95% CI: 16.2–37.8, *I*^2^ = 88%; Fig. 6*E*); psychiatric symptoms were observed in 30.8% (95% CI: 17.2–46.2, *I*^2^ = 81%; Fig. 6*F*); dysarthria were observed in 25.5% (95% CI: 13.3–39.8, *I*^2^ = 71%; Fig. 6*G*); and asymptomatic cases were observed in 23.5% (95% CI: 12.7–36.1, *I*^2^ = 86%; Fig. 6*H*). Egger's linear regression analysis indicated no significant publication bias (*p* > 0.05). Except for headache, cognitive impairment, and speech disorder phenotypes, which showed moderate heterogeneity, the remaining outcomes exhibited high heterogeneity. Sensitivity analysis using the “leave-one-out” method confirmed that the combined results were robust and unaffected by the exclusion of any individual study.

## Discussion

This integrative analysis identifies *SLC20A2* and *MYORG* as predominant genetic contributors to PFBC, with distinct neuropsychiatric manifestations (cognitive deficits, psychiatric symptoms) representing hallmark clinical features. The mid-adult disease onset pattern reinforces PFBC's characterization as a neurodegenerative disorder with presymptomatic calcification progression. These findings position *SLC20A2* and *MYORG* variants as critical molecular markers while underscoring the need to disentangle mechanisms underlying its striking phenotypic heterogeneity.

In the keyword network analysis, *SLC20A2* exhibited the largest node area among all related genes. Moreover, in the meta-analysis, variants associated with *SLC20A2* showed the highest detection rates, and patients carrying these variants had significantly higher TCS scores. The pathogenic effect of *SLC20A2* variants remains unclear, but one possible explanation is that variants in *SLC20A2* disrupt the normal function of PiT-2, leading to phosphate homeostasis imbalance. This causes excessive phosphate absorption by brain cells, particularly astrocytes, resulting in calcium phosphate crystal deposition in brain tissue. These calcifications can accumulate in various regions of the brain, including the basal ganglia (Wallingford et al., 2017; Zhang et al., 2023).

The *XPR1* gene also contributes to pathogenesis by directly impairing phosphate transport in brain cells and inhibiting the storage of phosphate by suppressing the intracellular accumulation of polyphosphates (Mailer et al., 2021; Xu et al., 2023). In addition, in our meta-analysis we observed that *XPR1* was associated with lower TCS scores. The differential impact of *XPR1* and *SLC20A2* variants on calcification severity may stem from their distinct roles in phosphate homeostasis. Although both genes encode phosphate transporters, *SLC20A2* (PiT-2) mediates constitutive cellular phosphate uptake across the blood–brain barrier and the choroid plexus (Jensen et al., 2016), thereby directly affecting the clearance of phosphate from the cerebrospinal fluid. Consequently, complete loss-of-function mutations in *SLC20A2* may result in systemic phosphate dysregulation, predisposing patients to widespread calcification. In contrast, *XPR1* primarily regulates phosphate efflux via its plasma membrane localization, functioning as a “phosphate sensor” that modulates cellular output through a feedback mechanism (Legati et al., 2015; Anheim et al., 2016). This functional specialization suggests that *XPR1* variants may retain partial phosphate efflux capacity through residual transporter activity or compensatory pathways, thereby potentially limiting the calcification burden.

In addition to the phosphate metabolism pathway, some studies suggest that the pathogenic effects of variants in other genes may be related to damage to the NVU. The NVU, as the smallest functional unit of the brain, plays a crucial role in regulating cerebral blood flow and maintaining the integrity of the BBB. Dysfunction of the NVU can disrupt normal exchanges of substances between the blood and brain parenchyma. If the BBB is compromised, increased permeability may allow high concentrations of phosphate in the blood to infiltrate the brain, with *PDGFRB*, *PDGFB*, *MYORG*, and *JAM2* genes potentially affecting BBB integrity (Xu et al., 2023). The dominant genes *PDGFB*/*PDGFRB*, which function as a ligand–receptor pair, help maintain neurovascular unit stability by regulating pericyte recruitment; the associated calcification phenotype may be related to local BBB disruption in the basal ganglia (Keller et al., 2013; Nicolas et al., 2013b). In contrast, the recessive genes *MYORG* and *JAM2* impair the BBB through different mechanisms: *MYORG* regulates the astrocyte-mediated transport of endothelial tight junction proteins (Yao et al., 2018), while *JAM2*, being closely associated with tight junctions, directly maintains the integrity of the paracellular barrier (Yang et al., 2025). It is noteworthy that both “NVU” and “PiT-2” also appear as important nodes in the keyword network, marking significant directions for future research.

According to the results of the meta-analysis, the prevalence of cognitive disorders among PFBC patients was found to be the highest (45.3%) among all clinical phenotypes, followed by psychiatric disorders (30.8%). In the keyword network, “dementia” was also identified as an important node. One possible explanation for this is that excessive extracellular phosphate can lead to tissue toxicity (Hong et al., 2015) and neuroinflammation (Brown, 2020), which in turn damages surrounding brain tissue and impairs normal brain functions, causing common neurological and psychiatric symptoms in PFBC patients, such as memory loss, mood swings, and behavioral abnormalities (Suescun et al., 2019; Dunn et al., 2020). Additionally, “Parkinsonism” was also an important node in the keyword network. Although our results show that the prevalence of movement disorders was relatively low, the majority of studies in the meta-analysis reported on the occurrence of movement disorders, making this an important focus in PFBC research. Movement disorders may arise from motor neuron damage, abnormal neural conduction, or lesions in specific brain regions caused by brain calcification, especially when neural pathways between the basal ganglia and the cerebral cortex are affected. While this symptom is relatively rare among PFBC patients, when present, it significantly impacts the quality of life. Therefore, movement disorders remain an important area of concern in clinical research on PFBC and should be prioritized in early screening and clinical evaluation of the disease.

It is also worth noting that the meta-analysis observed that approximately one-fifth of PFBC patients were asymptomatic, while another one-fifth reported headaches, which is consistent with the rate of asymptomatic cases reported in a 2015 systematic review (Tadic et al., 2015). The genetic features of PFBC exhibit incomplete penetrance, meaning not all individuals carrying pathogenic variants will present with clinical symptoms. This incomplete penetrance may be related to variant type, the functional impact of the variants, and the timing of gene expression. Additionally, with advancing age, PFBC symptoms may gradually manifest, particularly in the context of neurodegeneration. Early carriers may not exhibit noticeable clinical signs due to their younger age or early physiological stage. As they age, the cumulative effects of brain calcification and neurodegenerative changes may promote the onset of clinical symptoms. Lastly, gene interactions, compensatory mechanisms, and gene–environment interactions may also influence the phenotypes of variant carriers. Certain carriers may have genetic interactions that are not yet fully understood, which could partially or fully compensate for the negative effects of the pathogenic variant, thereby preventing the appearance of symptoms. The heterogeneity of PFBC clinical phenotypes presents a challenge for clinical diagnosis and disease screening. Some asymptomatic variant carriers may only develop symptoms later due to specific environmental or physiological triggers, emphasizing the need for more research to support early screening and monitoring for PFBC.

In most of the outcomes from the meta-analyses, significant statistical heterogeneity was observed between studies. This variability may be attributed to differences in study populations, as the ratio of sporadic to familial cases varies across studies. Additionally, methodological heterogeneity in gene sequencing protocols contributes to the variability. Whole genome sequencing (WGS) method is more robust and less prone to missing variants compared with whole exome sequencing (WES; Belkadi et al., 2015). This suggests that future research should prioritize WGS when possible. The Baujat plot identified the study by Chen et al. in 2019 (Chen et al., 2019) as the primary source of heterogeneity, as it reported lower variant detection rates for *PDGFRB* compared with other studies. This study employed a retrospective cohort design, categorizing patients into a “systemic group” and a “nonsystemic group.” The “systemic group” consisted of 37 probands diagnosed at the Department of Neurology, Second Affiliated Hospital of Zhejiang University School of Medicine, while the “nonsystemic group” comprised 236 probands with cerebral calcification identified by imaging at other study centers. Patients with PFBC in the systemic group were ultimately diagnosed through outpatient clinics due to the presence of neurological symptoms, whereas those in the nonsystemic group were identified with calcification through imaging examinations and did not necessarily have neurological symptoms. It is possible that by including more asymptomatic individuals, this has expanded the study population to a certain extent, thereby introducing heterogeneity. It should be noted that, in addition to study design, the ratio of sporadic to familial cases included may also contribute to the high heterogeneity. For example, in the included studies, the research by Gebus et al. (Gebus et al., 2017) consisted solely of sporadic cases, which significantly differed from other studies (Table 2). Sporadic cases may be subject to substantial selection bias at the time of inclusion, with some sporadic patients potentially being overlooked due to atypical symptoms. For this reason, that study became the primary source of heterogeneity in the meta-analysis of *SLC20A2* detection rates.

Our study offers several advantages. First, it is the first meta-analysis to focus on the variant detection rates of various genes and the prevalence of different phenotypes in brain calcification patients. Additionally, we incorporated bibliometric perspectives by quantitatively exploring the characteristics and trends of the studies, and we used a random-effects model to combine the relevant outcomes. Second, we conducted a systematic search of databases and comprehensively included all 18 studies that met the inclusion criteria, all of which exhibited high methodological quality. Despite the high heterogeneity in some outcomes, no significant publication bias was detected between studies, and sensitivity analysis suggested robust results. Lastly, our bibliometric analysis was based on the WOSCC database, which includes complete citation networks for high-quality literature, ensuring the data's quality and authority (Wang and Waltman, 2016; Yeung, 2019).

However, there are several limitations to our study. First, although we used various methods, such as the Baujat plot and “leave-one-out” analysis, to explore the sources of heterogeneity, the exact sources of heterogeneity between studies have not been fully elucidated. Due to the limited number of studies included for each outcome, we were unable to perform meta-regression for further investigation. Additionally, some early studies did not differentiate between primary and secondary brain calcification, and there is a lack of studies from the Arabian Peninsula and Africa. A higher representation of affected individuals in the studies may introduce selection bias, making the combined results less generalizable to other populations. Secondly, certain outcomes were based on a limited number of studies, such as those reporting *MYORG* variant detection rates and the prevalence of dysarthria phenotypes, which were derived from only three studies, potentially compromising the accuracy of the results. Furthermore, as *JAM2* and *CMPK2* were discovered later, no studies meeting the inclusion criteria were available for these genes. Earlier identified genes, such as *SLC20A2*, may introduce estimation bias due to the relatively higher number of reports, leading to higher variant detection rates and TCS scores. Some potential PFBC pathogenic genes have not yet been discovered, and the detection rates of these undiscovered genes in the calcification population cannot be determined. Finally, due to design limitations, we were unable to compare calcification scores and the incidence rates of asymptomatic patients with age, despite evidence suggesting that the clinical expressivity of calcification correlates with age.

Our study has significant clinical implications. First, it provides a deeper understanding of the pathogenic mechanisms and research trends associated with PFBC, offering new insights into the molecular mechanisms of human brain function and neurodegenerative diseases. Clinically, our findings have implications for diagnosis and genetic counseling and provide valuable reference points for assessing prognosis and treatment outcomes. Genetic screening can be used to aid the diagnosis of brain calcification patients, in conjunction with common clinical phenotypic features of PFBC, improving diagnostic accuracy and enhancing patients’ quality of life. Future research should focus on variants related to *JAM2*, *CMPK2*, and *NAA60*, using more clinical data for validation, especially as the pathogenic effect of *CMPK2* on PFBC has yet to be independently replicated by other research groups. Further studies should also aim to expand the sample size and geographic scope, explore other potential pathogenic genes and variants, and analyze the associations and differences between different genotypes and phenotypes. Additionally, although current PFBC-related research is concentrated in China and France, it is essential to investigate pathogenic gene variants in brain calcification patients from other regions worldwide. Collaborative efforts between regional research institutions are needed to promote global research in this field. Finally, considering that current research focuses primarily on basic medical disciplines, and the keyword network shows a higher focus on “case report” type studies, future research could explore individual cases to identify potential therapeutic targets and emphasize clinical translational significance. The highly cited literature summarized in our study can provide valuable guidance.

### Conclusion

In PFBC patients, the *SLC20A2* and *MYORG* genes exhibit higher variant detection rates and tend to have higher TCS scores. Additionally, cognitive impairment and psychiatric symptom phenotypes have a higher prevalence in PFBC patients compared with other phenotypes, with ∼23.5% of patients remaining asymptomatic. Research on the genetic variants associated with PFBC has generated several hot topics. Institutions and researchers from different countries need to establish connections for more in-depth studies on the pathogenic mechanisms, clinical translation, and treatment of PFBC.

## Data Availability

The data that support the findings of this study are available from the corresponding author on reasonable request.

## Synthesis

Reviewing Editor: Karl Herrup, University of Pittsburgh School of Medicine Decisions are customarily a result of the Reviewing Editor and the peer reviewers coming together and discussing their recommendations until a consensus is reached. When revisions are invited, a fact-based synthesis statement explaining their decision and outlining what is needed to prepare a revision will be listed below. The following reviewer(s) agreed to reveal their identity: Martha Garcia-Ramirez.

The revised work has addressed the basic concerns of the reviewers and the editor.
